# Development of DNA markers for assisted selection of cassava resistant to cassava mosaic disease (CMD)

**DOI:** 10.1270/jsbbs.24046

**Published:** 2025-04-04

**Authors:** Hiroki Tokunaga, Pham Thi Nhan, Pham Thi Huong, Nguyen Hai Anh, Le Thi Mai Huong, Truong Minh Hoa, Nguyen Thi Huyen Trang, Nguyen Ba Tung, Cu Thi Le Thuy, Xiaofei Zhang, Motoaki Seki, Le Huy Ham

**Affiliations:** 1 Tropical Agriculture Research Front, Japan International Research Center for Agricultural Sciences, Ishigaki, Okinawa 907-0002, Japan; 2 Plant Genomic Network Research Team, RIKEN Center for Sustainable Resource Science, Yokohama, Kanagawa 230-0045, Japan; 3 Hung Loc Agricultural Research Center (HLARC), Thong Nhat 810000, Vietnam; 4 International Laboratory for Cassava Molecular Breeding (ILCMB), National Key Laboratory for Plant Cell Biotechnology, Agricultural Genetics Institute (AGI), Hanoi 100000, Vietnam; 5 Cassava Program, International Center for Tropical Agricultural (CIAT), Hanoi 100000, Vietnam; 6 Department of Plant Sciences, University of California, Davis, CA 95616, USA; 7 Cassava Program, International Center for Tropical Agriculture (CIAT), Km 17, Recta Cali-Palmira Apartado Aéreo 6713, Cali, Colombia; 8 Plant Epigenome Regulation Laboratory, RIKEN Cluster for Pioneering Research, 2-1 Hirosawa, Wako, Saitama 351-0198, Japan; 9 Kihara Institute for Biological Research, Yokohama City University, 641-12 Maioka-cho, Totsuka-ku, Yokohama, Kanagawa 244-0813, Japan

**Keywords:** cassava, cassava mosaic disease, marker-assisted selection, *CMD2*, MePOLD1

## Abstract

Cassava is an important staple crop in tropical and subtropical regions. Cassava mosaic disease (CMD) is one of the most dangerous diseases affecting cassava production in Africa. Since the first reported in Southeast Asia in 2015, the CMD prevalence has become a concern in Southeast Asia. To combat it, CMD resistance has been introduced from African cassava into Asian elite cultivars. However, efficient DNA markers for the selection of CMD resistance are not available. The *CMD2* locus confers resistance to African cassava mosaic virus via non-synonymous substitutions in the DNA polymerase δ subunit 1 gene (*MePOLD1*). Here, we developed DNA markers to identify the mutations providing the resistance. We examined the association between the resistance score in CMD-infected fields and the genotypes of hybrids of CMD-resistant and ‑susceptible Asian lines. Our study provides powerful tools to the global cassava breeding community for selecting CMD resistant cassava.

## Introduction

Cassava (*Manihot esculenta* Crantz, Euphorbiaceae) is an important staple crop grown in the tropics and subtropics (FAO 2021, Howeler *et al.* 2013). Africa produces the most, but the cultivation area has been increasing in Southeast Asia, not only for food but also for biofuel and starch (Howeler 2012, Marx 2019). However, the expansion of area and crop transportation has allowed the invasion and spread of new pests and diseases (Tokunaga *et al.* 2018).

Cassava production is threatened by two dangerous diseases: cassava mosaic disease (CMD) and cassava brown streak disease (Tokunaga *et al.* 2018). CMD has already spread to Africa, India, Sri Lanka, and Southeast Asia, but the latter is still confined to Africa. CMD is causing growing concern in Southeast Asia. Cambodia was the first country to report an outbreak of CMD, in 2015, since when the disease has rapidly invaded neighboring Vietnam, Thailand, Laos, and China (Uke *et al.* 2018, Wang *et al.* 2016, 2019, https://doi.org/10.1094/PDIS-09-20-1868-PDN). As CMD is a new disease in Southeast Asia, all cultivars in the region are susceptible, making it difficult to control (Minato *et al.* 2019, Uke *et al.* 2022). CMD is caused by viruses of the genus *Begomovirus* (*Geminiviridae*), a cassava mosaic begomovirus (CMB). In Africa and Asia, at least 10 known species of begomoviruses cause CMD; in Southeast Asia, Sri Lankan cassava mosaic virus (SLCMV) is prevalent at present (Saunders *et al.* 2002, Tokunaga *et al.* 2018, Uke *et al.* 2022, Zerbini *et al.* 2017, Zhou *et al.* 1997).

In Africa, utilizing genetic resources resistant to CMBs has been considered the most useful approach to controlling CMBs. Genetic mapping studies have discovered three loci (*CMD1*, *CMD2*, *CMD3*) that can confer resistance to the African cassava mosaic virus (Akano *et al.* 2002, Fregene and Puonti-Kaerlas 2001, Okogbenin *et al.* 2012, Rabbi *et al.* 2014). *CMD1*, derived from the wild cassava species *Manihot glaziovii*, is a recessive and polygenic locus, meaning that multiple genes are required to confer resistance. *CMD2* is a dominant and putative single genetic locus discovered among West African landraces by the International Institute of Tropical Agriculture (IITA) (Akano *et al.* 2002). It provides stable resistance in many cultivars, making it a highly valuable genetic resource for breeding programs in Africa. *CMD3* is a relatively new CMD-resistant QTL, discovered in the Nigerian cultivar TMS97/2205. It confers high resistance through synergistic effects with *CMD2* (Okogbenin *et al.* 2012).

Breeding projects have been initiated in Vietnam to develop CMD-resistant cultivars (Thuy *et al.* 2021, Uke *et al.* 2022, Vu *et al.* 2020). The projects use *CMD2*-type genetic resources introduced by the International Center for Tropical Agriculture (CIAT) and IITA to introgress *CMD2* into Asian elite cultivars (Thuy *et al.* 2021). However, screening takes a significant amount of time and cost and has led to a desire for DNA marker selection to reduce both. The SNPs markers S12_7926132 and S14_4626854 are used for selecting *CMD2* (Codjia *et al.* 2022, Rabbi *et al.* 2022), but recent field surveys showed that their utility is affected by the genetic background of the parents, and they are not always effective for selecting CMD-resistant plants from the diverse population pool (Thuy *et al.* 2021).

*CMD2* resistance is provided by mutations in the DNA polymerase δ subunit 1 gene (*MePOLD1*) (Lim *et al.* 2022), and *CMD2*-type-resistant lines carry one of three distinct single nucleotide substitutions on *MePOLD1* that cause a V528L, G680V, or L685F amino acid substitution near the active center of MePOLD1. However, DNA markers to distinguish these mutations have not been established, and the effect of the mutations against SLCMV has not been tested. SNPs markers S12_7926132 (Chromosome12:8782433, *Manihot esculenta* v8.1) and S14_4626854 (Chromosome12:16624313) are located 120 kbp and 7.7 Mbp, respectively, away from *MePOLD1* (Manes.12G077400|Chromosome12:8903764..8926251), so occasional recombination between the locus and these markers is possible. The objective of this study was to develop DNA markers to support simple methods to identify resistance-gene-carrying lines and verify the gene’s effect. We determined the DNA sequence of the *POLD1* gene of CMD-resistant cultivars introduced into Vietnam by CIAT and IITA, and developed a dCAPS analysis method and a Kompetitive Allele Specific PCR (KASP) assay based on the nucleotide sequence information.

## Materials and Methods

### DNA sequencing

Genomic DNA was extracted from leaves with a DNeasy Plant Mini Kit (Qiagen, 69104), following the manufacturer’s instructions. Target regions were amplified by PCR with KOD FX Neo polymerase (Toyobo, KFX-101) using the primers MePOLD-F1/R1 (Supplemental Table 1). The amplified DNA was cloned into the pTA2 vector with a Target Clone Plus Kit (Toyobo, TAK201) and transformed into *E. coli* competent DH5a cells. The colonies were used as PCR-amplification templates with primers M13RV/M13M4 (Supplemental Table 1). The PCR products were treated with ExoSAP-IT PCR Product Cleanup Reagent (Thermo, 78201) and analyzed by Sanger sequencing with primers MePOLD-F1 for exons 13 and 14 of *MePOLD1*, MePOLD-F2 for exon 15, and MePOLD-R1 for exons 16, 17, and 18 by Macrogen Japan. The sequences were aligned against the *Manihot esculenta* v. 8.1 reference sequence from the Phytozome database (https://phytozome-next.jgi.doe.gov).

### dCAPS analysis

**Fig. 1.**
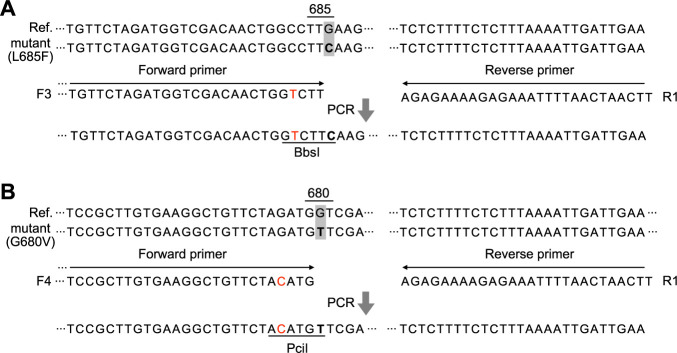
Design for dCAPS analysis. Primers designed to detect (A) L685F and (B) G680V. The DNA mutations conferring the amino acid substitution in lines HN4 (L685F mutant) and C-33 (G680V mutant) against the Reference sequence (Ref.) are indicated in bold type. Forward primers are designed to be complementary to the DNA of both Ref. and mutants but to contain 1 mismatch (in red). Analysis of L685F used primers F3 and R1, and that of G680V used F4 and R1. PCR with each primer set produces (A) the *Bbs*I site and (B) the *Pci*I site only when mutant genomic DNA is used as a template.

We designed dCAPS primers to detect the L685F mutation in the dCAPS Finder program (Neff *et al.* 2002). The forward primers were designed to create recognition sites for restriction enzymes within the PCR products, but only when a mutant allele is used as a template. We tried three forward primers, each possessing 1 or 2 nucleotide mismatches near the 3ʹ end (Supplemental Table 1, Supplemental Figs. 1A, 4A, 4B). We designed forward primer 4 to detect G680V in the dCAPS Finder program (Fig. 1B). The reverse primer is located ~230 bp from the forward primers and has no mismatches.

PCR amplification was performed with KOD FX Neo using genomic DNA extracted from leaves as a template and the primer sets listed in Supplemental Table 1; for L685F detection, forward primer 1, 2, or 3 and reverse primer 1; for G680V detection, forward primer 4 and reverse primer 1; for S12_7926132 detection, forward primer 5 and reverse primer 2. The thermal conditions were an initial 94°C for 2 min, followed by 35 cycles of 98°C for 10 s, 55°C for 30 s, and 68°C for 20 s. PCR samples were cleaned up by ethanol precipitation. Each PCR product was cut by restriction enzyme *Pci*I (NEB, R0655), *Bbs*I (NEB, R3539), *Bcc*I (NEB, R0704), *Eco*RI (NEB, R3101) or *Nco*I (NEB, R3193) according to the manufacturer’s manual and then electrophoresed in 3% (w/v) agarose gel (Nippon Gene Co., Agarose S) in Tris·Borate·EDTA buffer.

### KASP genotyping

We established a second genotyping method that relies on fluorescence-based selection by the KASP genotyping system (Primetech), which is used for large-scale breeding projects by CIAT and IITA (Thuy *et al.* 2021). To validate the accuracy of the KASP genotyping for the selection of G680V genotypes, we used DNA templates from the cassava breeding materials shown in Table 1. Because no cultivar has a homozygous mutation in *POLD1* (T/T genotype at G680), we created a plasmid carrying the T allele for the positive control.

We used KASP-TF v. 4.0 MM96/384 High ROX master mix (Primetech, KBS-1050-131) following the manufacturer’s instructions. Custom primers and probes were designed by the manufacturer’s platform (Primetech; Supplemental Table 1). The assay and fluorescence reading were carried out in a StepOnePlus Real-Time PCR System (Applied Biosystems) with an initial denaturation at 94℃ for 15 min, followed by touchdown PCR with an annealing temperature of 61℃ dropping by 0.6℃ per cycle over 10 cycles, and then 26 cycles of 94℃ for 20 s and 55℃ for 60 s. To create a SNP-positive control, we prepared plasmid DNA clones carrying nucleotide mutations at position G680 or L685: PCR amplification was performed with KOD FX Neo using genomic DNA extracted from the CMD-resistant line C-33 (G/T allele, heterozygous G680V) or the CMD-resistant line HN3 (G/C allele, heterozygous L685F, Table 1) as template and the primers MePOLD-F1 and MePOLD-R1 (Supplemental Table 1); the PCR products were cloned into vector pTA2, and plasmids carrying the mutation allele were selected among the obtained clones.

### Field evaluation of CMD resistance

The CMD-resistant C-33 was crossed to Asian cultivars KU50, HL-S12, and HL-S14 in 2020 in Lam Dong province, Vietnam (Tokunaga *et al.* 2022). Nine plants each of 20 hybrid lines were evaluated for CMD resistance in a field plot at Hung Loc Agricultural Research Center in 2021 and 2022. CMD-infected cuttings of HL-S14 were planted every 5 rows to promote infection. Disease symptoms were scored 3 months after planting according to the IITA general criteria, from 1 (no symptoms) to 5 (small curled leaves with severe chlorosis) (Hahn *et al.* 1980). Previous data on 40 CIAT lines with the same resistance origin as C-33 were analyzed (Thuy *et al.* 2021). For classification of S12_7926132 alleles, the 20 hybrid lines and three Asian cultivars were analyzed by dCAPS assay, and the CIAT 40 lines were analyzed through Intertek genotyping Platform in a previous study (Thuy *et al.* 2021).

## Results

### DNA sequence analysis

Genomic sequences of genetic resources introduced into Vietnam from CIAT in Colombia and IITA in Nigeria suggest that CMD resistance is due to V528L, G680V, or L685F substitutions near the active center of MePOLD1 (Lim *et al.* 2022), corresponding to exons 13–18 (Supplemental Figs. 1–3). Sequencing of exons 13–18 and the corresponding introns showed that Colombian lines C-33, C-36, CR100-9 (managed as C-30 in Vietnam), AR23-1 (C-41), CR27-20 (C-80), AR42-4 (C-48), and AR9-48 (C-97) harbor a heterozygous G680V mutation (Table 1, Supplemental Fig. 3); and that Nigerian lines IBA920057 (registered as HN2 in Vietnam, where “HN” = Hanoi), IBA972205 (HN3), IBA980505 (HN4), and IBA980581 (HN5) harbor a heterozygous L685F mutation (Table 1, Supplemental Fig. 3); our genetic resources harbored no V528L mutation (Supplemental Fig. 2). As far as we could ascertain, all of the resistant lines contain heterozygous mutations in MePOLD1, indicating that homozygous mutations may be detrimental (Lim *et al.* 2022). The tolerant cultivar TMEB419 (HN1), which shows mild CMD disease symptoms with no effect on growth and yield, had no G680V/L685F substitutions (Table 1). The Asian cultivars KU50, HL-S12, and HL-S14 and the recently found Vietnamese cultivars VN19-442, VN19-773, VN19-1556, and VN19-1050 did not have any non-synonymous mutation in the active center of MePOLD1 (Table 1). We developed our DNA markers to detect mutant alleles causing the G680V and L685F substitutions.

### Design of dCAPS tool

**Fig. 2.**
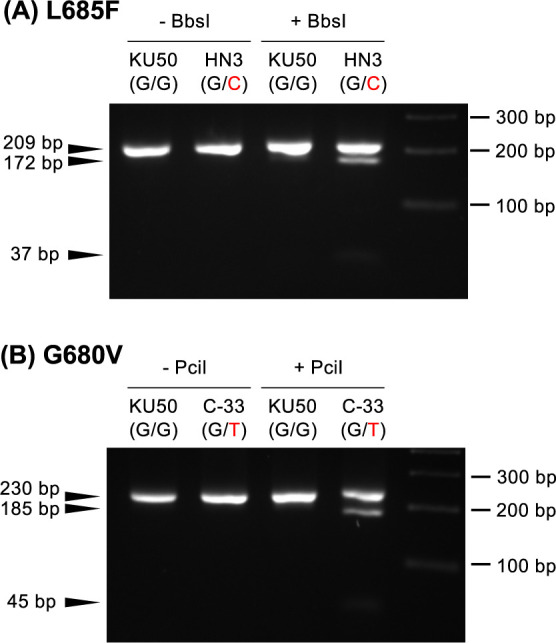
Examples of the use of dCAPS markers. Electrophoresis patterns of dCAPS marker analysis of (A) L685F and (B) G680V. PCR products amplificated from the KU50 (wild type) template could not be cleaved by either (A) *Bbs*I in L685F or (B) *Pci*I in G680V. When lines HN3 (heterologous L685F, G/C) and C-33 (heterologous G680V, G/T) were used as templates, the restriction enzymes digested the PCR products. It is difficult to visually judge the presence of the shorter fragments (37 bp in A, 45 bp in B); therefore, the presence or absence of the longer digested fragment (172 bp in A, 185 bp in B) is used to judge the presence or absence of the mutations.

With forward primers 1 and 2, the PCR products amplified from the mutant-allele templates were not cleaved by the restriction enzyme. Sequencing showed that the designed mismatches in the primers had been replaced with the template sequence, eliminating the recognition sites (Supplemental Fig. 4C). This can be attributed to the 3ʹ→5ʹ exonuclease activity of the DNA polymerase that we used for this assay and the elimination of the mismatched bases of the primers during PCR. Forward primer 3 created a *Bbs*I recognition site in the PCR product of the mutant allele (Fig. 1A), and no unexpected nucleotide incorporation occurred (Supplemental Fig. 4C). *Bbs*I digested the 209-bp PCR product from the mutant allele into 172- and 37-bp fragments (Fig. 2A). The 37-bp fragment was just distinguishable in 3% agarose gel. PCR using forward primer 4 amplified a 230-bp product that was digested by *Pci*I to 185- and 45-bp fragments (Fig. 2B).

### KASP genotyping

**Fig. 3.**
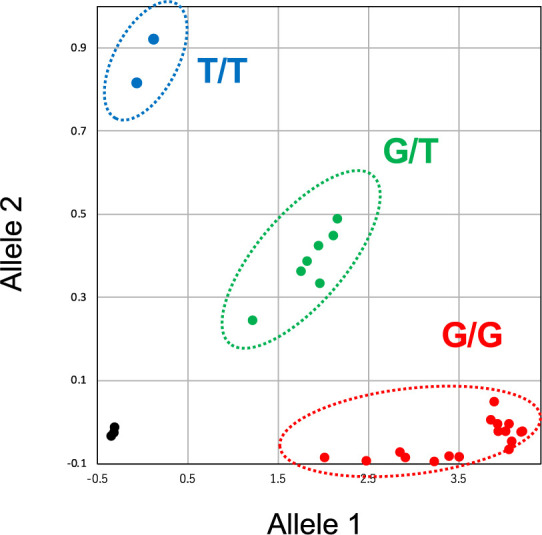
Allelic discrimination plot for *POLD1*-G680V mutation in KASP genotyping assay. FAM fluorescent signal values are plotted on the *x*-axis and VIC fluorescent signal values on the *y*-axis. The circle along the *x*-axis shows G/G genotypes and the circle along the *y*-axis shows T/T genotypes, and the circle in middle showed G/T genotypes. Black dots indicate no-template control.

The amplification signals from the homozygous T/T genotypes (VIC fluorescence) were gathered near the *y*-axis, those of the homozygous G/G genotypes (FAM fluorescence) near the *x*-axis, and those of the heterozygous G/T genotypes between the two homozygous clusters (Fig. 3). These results corresponded perfectly with those of the sequencing. On the other hand, the KASP assay for L685F genotyping did not separate the signals (Supplemental Fig. 5), probably owing to low GC contents (33.3%) of the primer sequence near the allele position. These results show that the KASP method is suitable for selecting G680V genotypes.

### Association between *POLD1* genotype and CMD resistance

**Fig. 4.**
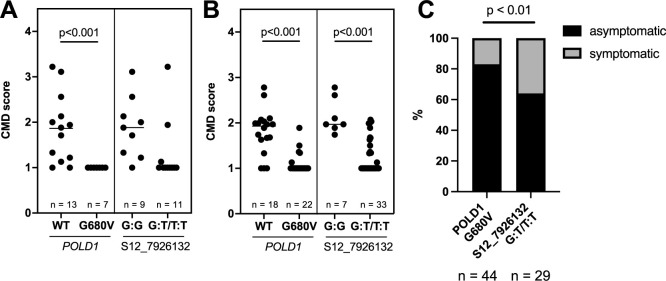
Association between *POLD1* genotype and CMD severity. CMD scores of (A) Asian elite cultivars × C-33 hybrids and (B) South American cultivars × CIAT hybrids in the CMD infection field survey. CMD symptoms were scored from 1 to 5 at 3 months after planting. The CMD score are shown when grouped by the *POLD1* genotype (left side in A and B) and SNP types of S12_7926132 (right side in A and B). Bars indicate median CMD score. Differences are significant by Mann–Whitney *U*-test. (C) Percentages of symptomatic and symptomless plants in populations classified by the commonly used SNP marker for *CMD2* selection (S12_7926132) and by the dCAPs marker detecting the *POLD1*-G680V mutation. The difference is significant by Fisher’s exact test.

The genotypes of the 20 hybrid lines bred by crossing C-33 with Asian cultivars were classified with the dCAPS marker. Three months after planting, 85% (11/13) of wild-type (WT) *POLD1* genotypes had CMD symptoms (Fig. 4A, left), but all lines (7/7) with the G680V mutation in *POLD1* had no symptoms (CMD score 1), even under high CMD pressure. The CMD score was significantly lower in G680V genotypes than in WT (Fig. 4A, left), showing that the DNA markers we developed can efficiently select lines resistant to CMD. By contrast, in the case of classifications by the SNP marker S12_7926132, the G:T/T:T group had a non-significantly lower score than the G:G group (Fig. 4A, right).

dCAPS genotyping of the 40 progeny bred in CIAT identified 22 lines with the G680V mutation, 18 with the WT genotype, and none with the L685F mutation (Fig. 4B, left). Of the WT genotypes, 83% (15/18) had CMD symptoms and 17% (3/18) had no symptoms. On the other hand, of the G680V genotypes, 23% (5/22) had mild CMD symptoms (<2) and 77% (17/22) had no symptoms. The G:T/T:T group identified by S12_7926132 had a significantly lower score than the G:G group in this genetic pool (Fig. 4B, right). Overall, the *POLD1*-G680V genotypes had significantly lower CMD symptom scores than the WT genotypes in both genetic pools (Fig. 3A, 3B). Thus, we confirmed that the DNA marker tools, which can detect the *POLD1* mutation, effectively distinguish CMD-resistant and -susceptible cassava lines from diverse populations.

Of the 60 lines comprising the 20 hybrids and the CIAT 40 lines, 71.8% (44/60) had the T:T and G:T SNPs at S12_7926132, indicating that S12_7926132 has low efficiency in eliminating CMD-sensitive lines. Of those lines, 64% of the lines with G:T/T:T were asymptomatic (Fig. 4C) and the other 36% were symptomatic. On the other hand, 83% of the G680V population were a symptomatic, clearly showing that the new DNA marker tools are more efficient for breeding CMD-resistant lines.

## Discussion

This research presents two types of DNA marker tools that can select CMD-resistant cassava in the hybrid breeding pool: dCAPS analysis and KASP genotyping. The dCAPS method requires only restriction enzyme treatment of PCR samples, eliminating the need for expensive detecting instruments. It can be used widely and is especially useful for small-scale breeding programs. The KASP genotyping assay requires more initial costs but can reduce the number of experimental steps and eliminate operator error. It can be powerful for large-scale breeding programs. This study evaluated the efficacy of DNA markers on a limited scale. Future research should test a broader range of genetic resources. These markers will be beneficial in practical cassava breeding programs to assess performance.

Two CMD-resistant cultivars, IBA972205 (HN3) and IBA980581 (HN5), have recently been registered in Vietnam. Both were introduced from IITA in Nigeria and have been distributed around Vietnam to control the CMD epidemic. Although they are stably resistant to CMD, they have not been widely accepted by farmers owing to their lower yield and starch content than the Asian elite cultivars. This lower performance might be due to differences in the breeding process between Asia and Africa and in the intended use of the cassava: in Africa, cassava is used primarily for food, while in Southeast Asia, it is used for industrial purposes, for which high yield and starch content are required (Malik *et al.* 2020). Breeders have now begun to transfer *CMD2* resistance into Asian cultivars.

As in previous works (Codjia *et al.* 2022, Hahn *et al.* 1980, Lim *et al.* 2022, Thuy *et al.* 2021), we evaluated susceptibility to CMD infection by visually scoring the disease symptoms in the field. Our field test conditions can be considered as enabling a high intensity of SLCMV transmission by whitefly (*Bemisia tabaci*). However, the intensity and timing of infection across all tested lines might not be uniform, so it’s not accurate at this stage to judge that lines with no CMD symptoms are truly resistant; some resistant or tolerant lines show initial disease symptoms, which disappear as the plants grow (Lim *et al.* 2022, Thuy *et al.* 2021). Some lines with the *POLD1* mutation showed mild symptoms (Fig. 4B). Future breeding projects will need to more accurately evaluate CMD resistance, considering its influence on tuber yield, not just disease symptoms, through tests in multiple years and fields.

SNPs markers S12_7926132 and S14_4626854 were already options for *CMD2* selection (Codjia *et al.* 2022, Rabbi *et al.* 2022). Their distant locations from *MePOLD1* make occasional recombination between the locus and these markers possible. A search for other markers in diverse populations may not be effective because the SNPs in *POLD1* were acquired only recently in West African farmers’ fields, and the resistance due to the *POLD1* mutations would not be associated with the SNPs already present in diverse cassava population. Only if the alleles of breeding parents are limited and confirmed can the SNP markers select resistant lines from subsequent generations, but they are not effective in the selection from diverse populations whose parents are uncertain. Therefore, a more effective DNA marker should be designed to recognize the base-substitution mutation in *POLD1*.

Other resistance sources should also be explored. For now, the CMD pandemic in Southeast Asia can be resisted by the use of *CMD2*, although overreliance on *CMD2* as the only resistance source is inadvisable (Lim *et al.* 2022). As Lim *et al.* also found, no plant was homozygous for the mutation in *MePOLD1* (Lim *et al.* 2022). As DNA polymerase δ (POLD1) plays a role in nuclear DNA replication, which is essential for fundamental biological functions, homozygous G680V and L685F mutations may cause severe growth inhibition. If so, even a heterozygous mutation may limit plant growth and agronomic traits. It will be necessary to verify the effects of amino acid substitution mutations in MePOLD1 on agronomic traits. Some Vietnamese cultivators do not have the *CMD2*-type mutations but still show CMD tolerance (Thuy *et al.* 2021), for which *CMD1* and *CMD3* could be candidates. Future research is required to evaluate sources of resistance other than *CMD2*.

## Author Contribution Statement

HT designed research, performed the experiments, analyzed data, and wrote the article. PTN, PTH, NHA, CTLT designed and conducted experiments. LTH, TMH, NTHT, NBT performed the experiments. NHA, XZ, MS, LHH designed research and contributed to revisions of the article.

## Supplementary Material

Supplemental Figures

Supplemental Table

## Figures and Tables

**Table 1.** Response of cassava lines and varieties on infection with SLCMV and their genotypes in POLD1

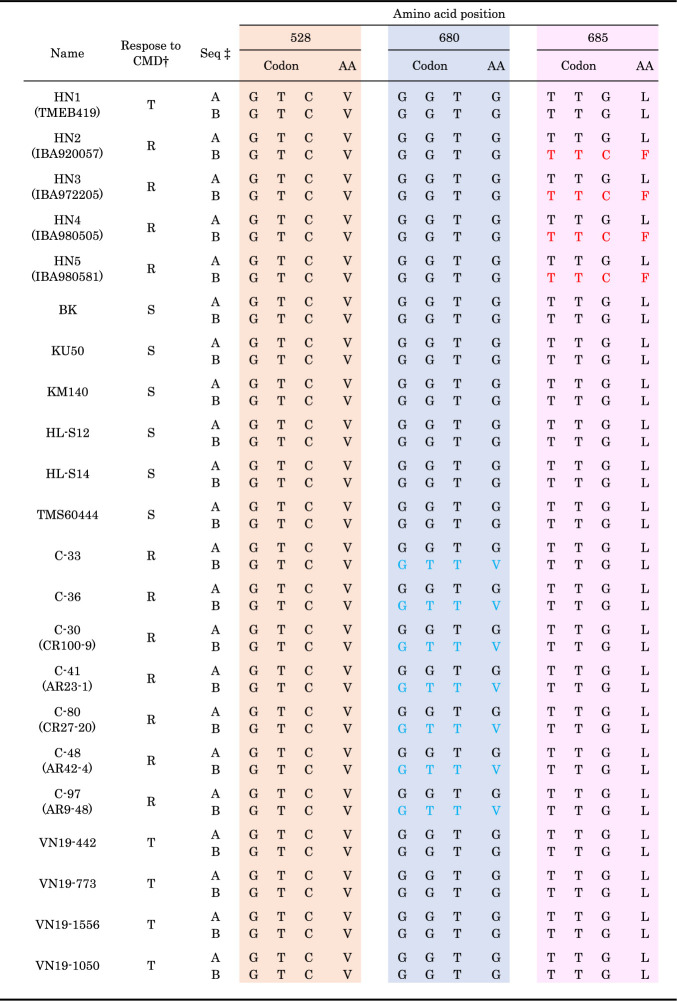

^†^ R: Resistant; T: Tolerant, S: Sensitive. Resistant indicates no CMD symptom even when cultivated in fields widespread with CMD. Tolerant indicates CMD symptoms but no or little effect on growth and yield. ^‡^ A and B in seq: 2 different patterns revealed by sequencing.
