# Supplementary material for: Development of DNA markers for assisted selection of cassava resistant to cassava mosaic disease (CMD)
Source: Breed Sci. 2025 Apr 4;75(2):111–8. doi: 10.1270/jsbbs.24046 (PMC12395196; doi:10.1270/jsbbs.24046)
Supplement: Supplementary file 1 — Supplemental Figures [file 75_111_s1.pdf]

Tokunaga et al./Breeding Science
doi: 10.1270/jsbbs.24046

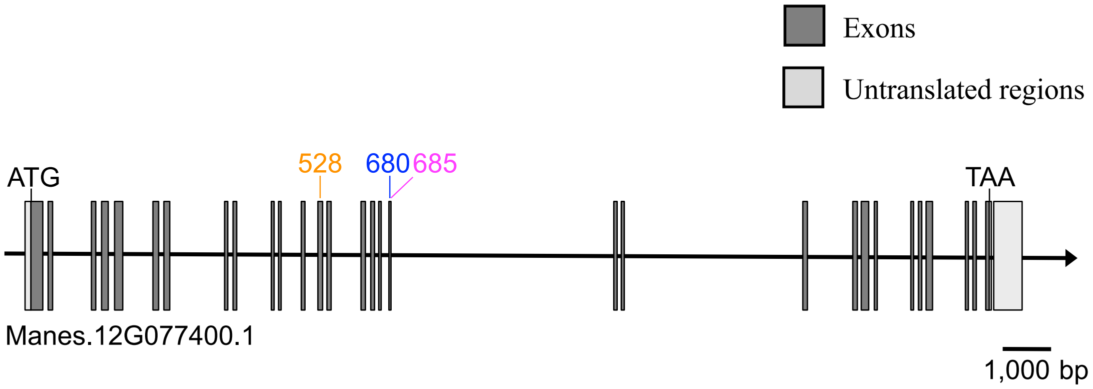

Supplemental Figure 1. Genomic structure of gene encoding DNA polymerase δ subunit 1 (*MePOLD1*)
Numbers indicate the positions of codons encoding amino acid substitutions V528L, G680V, and L685F.

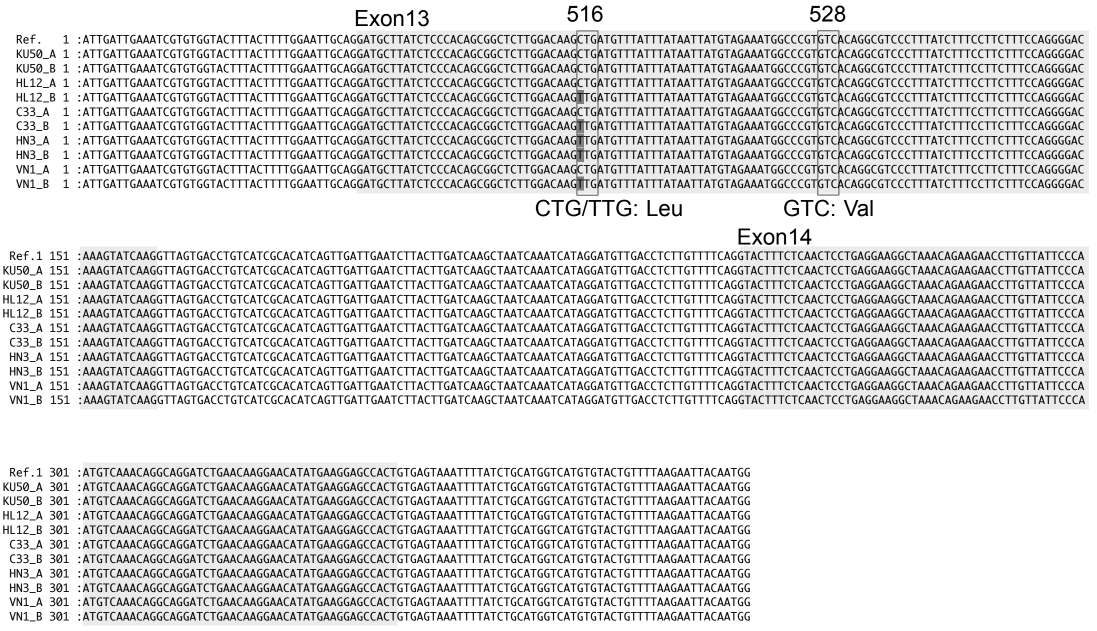

Supplemental Figure 2. Genomic DNA sequences of exons 13 and 14 in *MePOLD1*.
Alignment of genome sequences from Reference (Ref.) and Asian cultivars KU50, HL-S12 (HL12), C-33 (C33), HN3, VN19-442 (VN1). PCR products amplified from DNA extracted from each cultivar were cloned and sequenced. Owing to the heterozygous-diploid genome, the resulting clones contained different DNA sequences. Gray shading indicates exon regions. Numbers indicate positions of codons encoding Leu (516) and Val (528) of MePOLD1. A SNP at position 516 encodes a synonymous substitution. The reference sequence was taken from *Manihot esculenta* v. 8.1 in the Phytozome database.

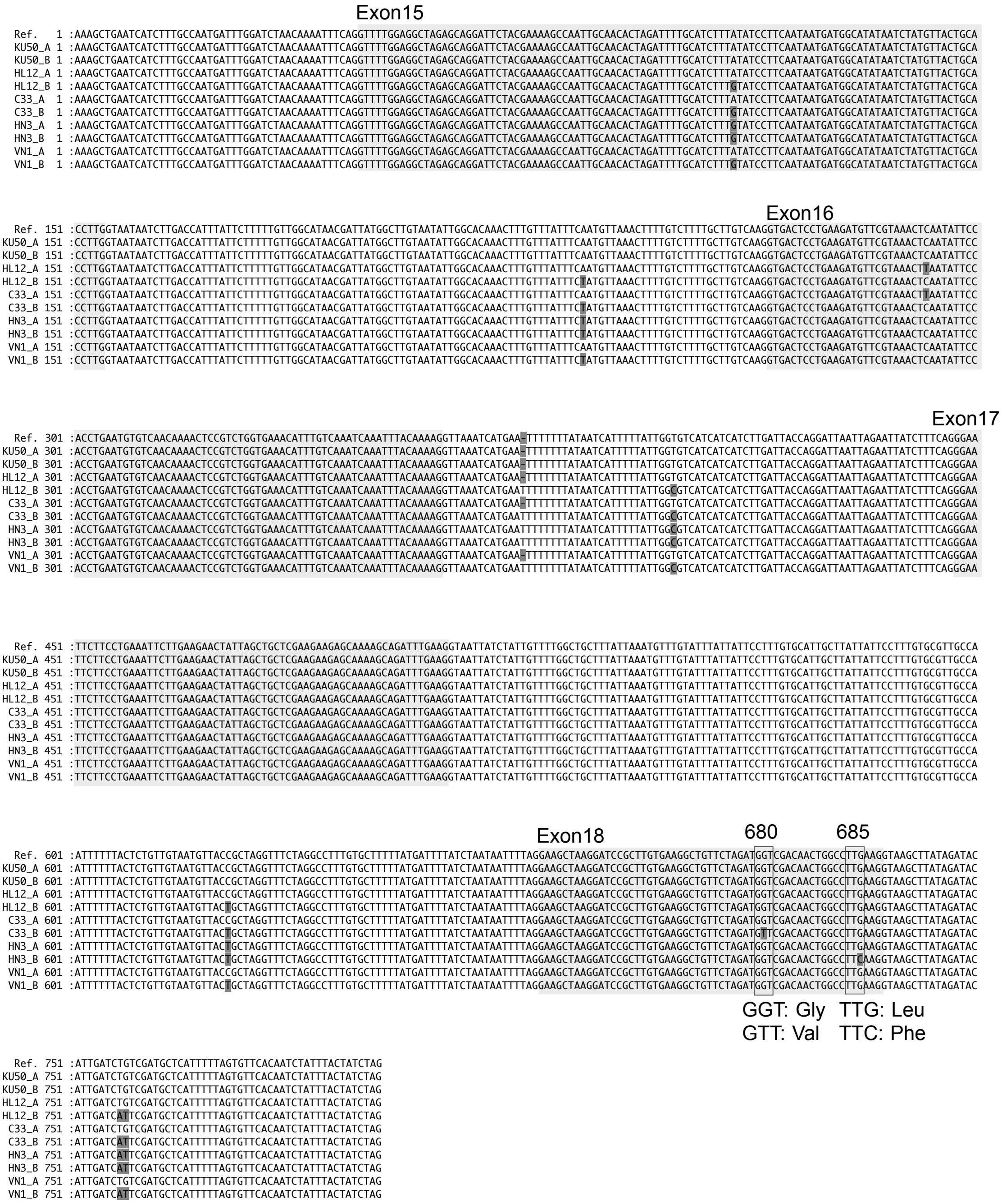

Supplemental Figure 3. Genomic DNA sequenced of exons 15–18 in *MePOLD1*.
Alignment of genome sequences from Reference (Ref.) and Asian cultivars KU50, HL-S12 (HL12), C-33 (C33), HN3, VN19-442 (VN1). PCR products amplified from DNA extracted from each cultivar were cloned and sequenced. Owing to the heterozygous-diploid genome, the resulting clones contained different DNA sequences. Gray shading indicate exon regions. Numbers indicate positions of codons encoding Gly (680) and Leu (685) of MePOLD1. The reference sequence was taken from *Manihot esculenta* v. 8.1 in the Phytozome database.

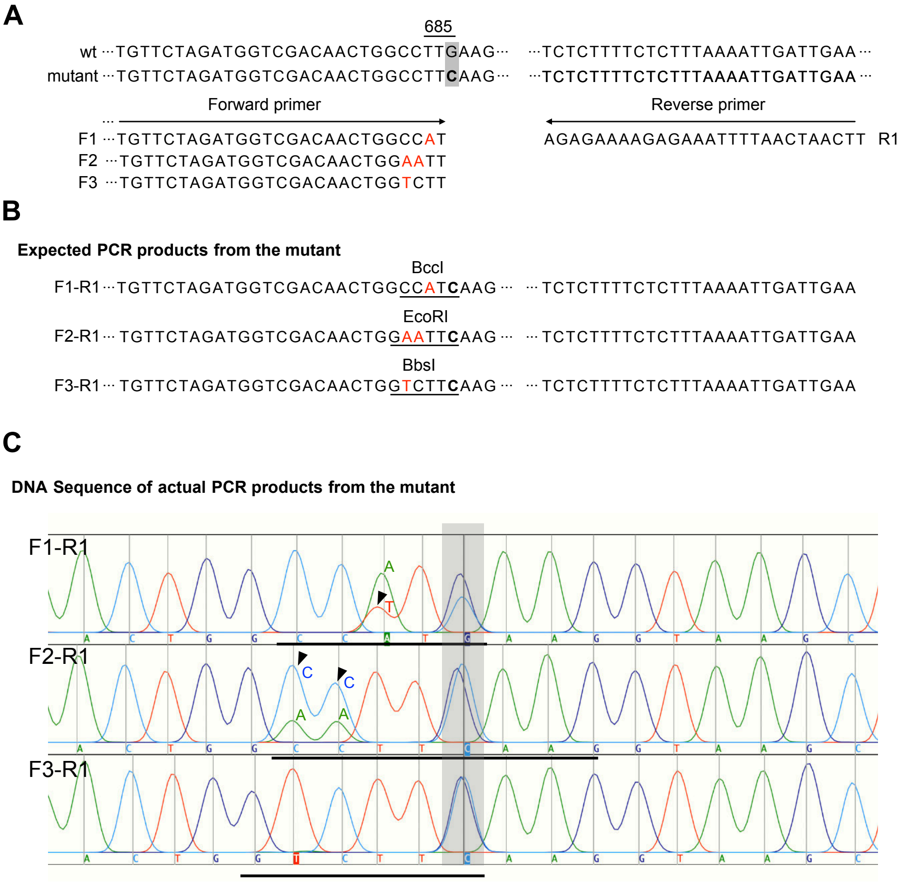

Supplemental Figure 4. Effect of position of mismatches in primers for dCAPS.
(A) Design of candidate primers to detect L685F. The DNA mutation conferring the amino acid substitution in the L685F mutant against the Reference sequence (Ref.) is indicated in BOLD type. Forward primers (F1, F2, F3) are designed to be complementary to the DNA of both Ref. and mutants but to contain 1 or 2 mismatches (in red). (B) DNA sequences of PCR products from the mutant DNA expected by using forward primers F1, F2, and F3 with reverse primer (R), producing the *BccI*, *EcoRI*, and *BbsI* sites, respectively, when the mutant genomic DNA is used as a template. (C) Chromatograms of directly sequenced PCR products amplified from L685F-mutant DNA using primers F1, F2, and F3. Underlines indicate recognition sites for the restriction enzymes. Arrowheads show unexpected nucleotides.

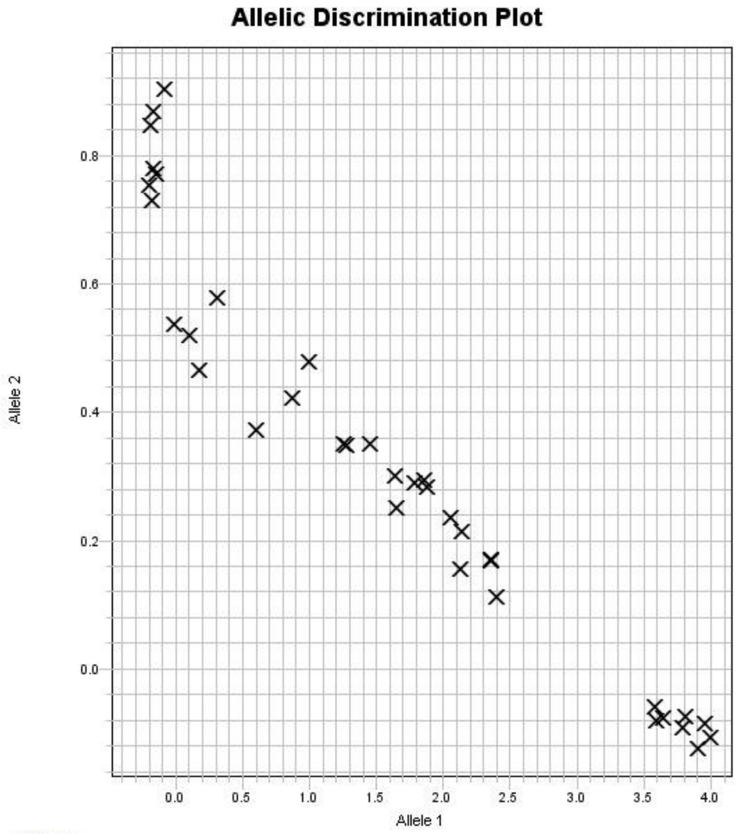

Supplemental Figure 5. Allelic discrimination plot for *POLD1-L685F* mutation using KASP genotyping assay.

FAM fluorescent signal values are plotted on the x-axis and VIC fluorescent signal values are plotted on the y-axis. The cross shows undetermined genotype.
