# Supplementary material for: Development of DNA markers for assisted selection of cassava resistant to cassava mosaic disease (CMD)
Source: Breed Sci. 2025 Apr 4;75(2):111–8. doi: 10.1270/jsbbs.24046 (PMC12395196; doi:10.1270/jsbbs.24046)
Supplement: Supplementary file 2 — Supplemental Table [file 75_111_s2.pdf]

Tokunaga et al./Breeding Science
doi: 10.1270/jsbbs.24046

Supplemental Table 1. Primer Sequence

For DNA sequencing

Identifier	Sequence (5' to 3')	
MePOLD-F1	TCTGTGGCCCTTTTTCCTAA	
MePOLD-R1	ATTTGAGGCCATCCACTTGA	
M13RV	GGAAACAGCTATGACCAT G	
M13 M4	GTTTTCCCAGTCACGAC	
MePOLD-F2	CTGCAAGTGCCACTATTTCAA	

For dCAPS analysis

Identifier	Sequence (5' to 3')	purpose
Forward primer #1	TTGTGAAGGCTGTTCTAGATGGTCGACAACTGGCCAT	To add BccI site for L685F detection
Forward primer #2	GATCCGCTTGTGAAGGCTGTTCTAGATGGTCGACAACTGGAATT	To add EcoRI site for L685F detection
Forward primer #3	GATCCGCTTGTGAAGGCTGTTCTAGATGGTCGACAACTGGTCTT	To add BbsI site for L685F detection
Forward primer #4	TAATAATTTTAGGAAGCTAAGGATCCGCTTGTGAAGGCTGTTCTACATG	To add PciI site for G680V detection
Reverse primer #1	TTCAATCAATTTTAAAGAGAAAAGAGA	Reverse primer for both G680V and L685F analysis
Forward primer #5	TCCTATTTTCCATGTTTCCACCCTCCCATG	To add NcoI site for S12_7926132 detection
Reverse primer #2	ATCTGCTGGAGAAGAATTACACCAAT	Reverse primer for S12_7926132 detection

For KASP genotyping

Identifier	Sequence (5' to 3')	purpose
pold1_G680V_common	TCCGCTTGTGAAGGCTGTTCTAGAT	
pold1_G680V_AllelexX	TACCTTCAAGGCCAGTTGTCGAC	AlleleX: G
pold1_G680V_AllelexY	TTACCTTCAAGGCCAGTTGTCGAA	AlleleY: T
pold1_L685F_common	TCCGCTTGTGAAGGCTGTTCTAGAT	
pold1_L685F_AllelexX	TACCTTCAAGGCCAGTTGTCGAC	AlleleX: G
pold1_L685F_AllelexY	TTACCTTCAAGGCCAGTTGTCGAA	AlleleY: C
